# Evolution of Microstructure and Properties of Air-Cooled Friction-Stir-Processed 7075 Aluminum Alloy

**DOI:** 10.3390/ma15072633

**Published:** 2022-04-02

**Authors:** Józef Iwaszko, Krzysztof Kudła

**Affiliations:** 1 Department of Materials Engineering, Faculty of Production Engineering and Materials Technology, Czestochowa University of Technology, 19 Armii Krajowej Ave., 42-200 Czestochowa, Poland; 2 Department of Technology and Automation, Faculty of Mechanical Engineering and Computer Science, Czestochowa University of Technology, 21 Armii Krajowej Ave., 42-200 Czestochowa, Poland; krzykudla@gmail.com

**Keywords:** friction stir processing, 7075 aluminum alloy, air cooling, jet cooling nozzle, microstructure evolution, hardness, tribological properties

## Abstract

A rolled plate of 7075 aluminum alloy was friction-stir-processed (FSP) with simultaneous cooling by an air stream cooled to −11 °C with a jet cooling nozzle. Two variants of air blowing were used: at an angle of 45° to the sample surface and at an angle of 90°. The reference material was a sample subjected to analogous treatment but naturally cooled in still air. The microstructural tests revealed strong grain refinement in all the samples, with higher grain refinement obtained in the air-cooled friction-stir-processed samples. For the naturally cooled samples, the average grain size in the near-surface area was 7.6 µm, while for the air-cooled sample, it was 1.4 µm for the 45° airflow variant and 3.2 µm for the 90° airflow variant. A consequence of the greater grain refinement was that the hardness of the air-cooled friction-stir-processed samples was higher than that of the naturally cooled samples. The improvement in abrasive wear resistance was achieved only in the case of the friction-stir-processed specimens with air cooling. It was found that the change in the air blowing angle affects not only the degree of grain refinement in the stirring zone, but also the geometrical structure of the surface. In all the samples, FSP caused redistribution of the intermetallic precipitates combined with their partial dissolution in the matrix.

## 1. Introduction

Friction stir processing (FSP) is a novel grain refinement technique and one of the most promising methods of modifying the microstructure and properties of engineering materials. This solid-state processing technique was developed by Mishra et al. [1,2], but the basic principles and idea of friction stir processing are derived from friction stir welding (FSW) technology [3]. The differences between FSP and FSW mainly concern their purpose because FSW technology is used to join materials [4,5], while FSP is employed to modify the microstructure of the material [6,7,8,9]. As with FSW technology, FSP uses a special cylindrical tool with a pin that rotates and plunges into the material to be processed, and then moves along designed paths. The nature and scope of changes occurring during FSP or FSW depend, among others, on the shape of the tool pin [10], the rotational speed [11] or the number of tool passes [12]. The friction of the tool against the surface of the modified material generates a large amount of heat, which makes the material plastic. The plasticized material flows to the back of the pin, where it is extruded and forged behind the tool, consolidated and cooled under hydrostatic pressure conditions [13]. FSP technology is also successfully used to eliminate defects and material loss in the sample [14] and to produce surface composites [15,16,17] or to modify the microstructure of composites [18,19]. The production of surface composites consists in introducing the reinforcing phase in the form of particles [20,21,22] or fibers [23] into the plasticized matrix. Currently, there are a number of solutions and methods allowing effective introduction of the reinforcing phase during FSP and the production of a surface composite [24]. For this purpose, the most common method is the groove method [25,26] or the hole method [27,28], less often used are direct friction stir processing [29,30,31], the sandwich method [23,32,33] or other solutions.

FSP technology is constantly evolving as new equipment and methodological solutions are constantly being developed, enabling more favorable changes to be obtained in the microstructure of the material, especially a higher degree of grain refinement. FSP is most often performed under conditions of natural cooling of the sample, i.e., in still air, and then the degree of grain refinement, and thus the properties of the material, are the result of the applied processing parameters. One of the newest trends in FSP technology is treatment with additional cooling in order to increase the temperature gradient in the material, and thus raise the cooling rate. The cooling rate of the material determines the degree of refinement of the microstructure, and therefore its mechanical properties; hence, the use of solutions increasing the cooling intensity is absolutely justified. The grain size affects not only the mechanical properties of the material [34], but also, for example, the corrosion resistance as smaller grains result in a lower value of the corrosion potential and the corrosion current [35]. A no less important aspect is the possibility of extending the tool life by reducing the amount of heat accumulated in the tool during FSP. The tool is exposed not only to abrasive wear, but also to high temperature resulting from friction against the surface of the material, as well as to cyclical changes in this temperature.

Currently, accelerated cooling is carried out by spraying coolant on the tool–workpiece interface [36,37], cooling the modified sample with compressed air [38,39] and conducting FSP on a sample immersed in a cooling agent [40,41] or intensive cooling of the sample before processing [42]. Special heat sinks with an internal cooling system are also utilized, in which the modified material is placed [43].

Assessment of the effect of additional cooling on the microstructure and material properties has been the subject of research by many research teams. For example, Luo et al. [12] analyzed the microstructure and properties of a material subjected to one-pass and two-pass submerged friction stir processing, using water as a coolant. In turn, Ai et al. [36] cooled samples of cast aluminum alloy A356 with a stream of water. The effect of cooling with water was greater grain refinement than in the analogous sample cooled with air, but at the same time, lower hardness of the alloy was obtained due to limitation of the growth of secondary-phase particles and their dissolution in the alloy matrix. Cooling with a stream of water was also used by Chen et al. [37]. In turn, Heidarpour et al. [43] used a water heat sink in surface modification of the AZ31 magnesium alloy. Derazkola et al. [44] tested three different coolants in the friction stir welding of aluminum AA3003 and A441 AISI steel sheet, namely, CO_2_, water and air, and found that the smallest grain size in the sample was obtained after using CO_2_, but simultaneously, the poorest joint with notable segregation at interface was formed. The water-cooled sample, in turn, was characterized by the greatest tensile strength, but at the same time, it was characterized by a lower hardness than in the case of cooling with CO_2_. In turn, Yazdipour et al. [45] employed a mixture of dry ice and ethanol to modify the Al5083 aluminum alloy. Analogous cooling was used by Satyanarayana et al. [46] in processing of the 6061 aluminum alloy. In turn, Moaref et al. [40] applied the water submerged processing technique in the friction stir processing of pure copper and a copper–zinc alloy, and Feng et al. [41] utilized the same method for treatment of the 2219-T6 aluminum alloy. Cooling of the AZ31B magnesium alloy by spraying liquid nitrogen was used by Ammouri et al. [42]. In turn, Alavi Nia [47] used a die made of copper with internal grooves for the flow of water and additional cooling by means of compressed air in the FSP of the AZ31 magnesium alloy.

By analyzing the literature data, it can be concluded that the application of accelerated cooling promotes refinement of the microstructure [48,49] and its homogenization [36,47] and usually improves the mechanical properties of the material [43,50]. However, it should be noted that some of the proposed solutions are difficult to implement in industry or their application requires additional operations and adaptation activities or even significant reorganization of the workplace. In the case of the most commonly used water cooling, it is necessary to dry the material after processing and remove the coolant from the working area, in addition to its management during and after FSP. Moreover, water as a coolant should not be used for materials that tend to corrode in an aqueous environment. Cryogenic cooling with liquid nitrogen makes it possible to obtain a higher cooling rate of the sample, but it is a problematic coolant owing to its intense evaporation in contact with a warmer material. The gas cloud formed in this case has insulating properties, which in turn reduces the effective cooling rate of the sample. The application of liquid nitrogen cooling is also troublesome because of the need to insulate all the components of the cooling system, which still does not completely exclude the risk of nitrogen gas formation in the cooling system. Another cryogenic coolant employed in FSP is dry ice, i.e., solid carbon dioxide. This material is usually utilized in the form of a mixture of dry ice and ethanol/methanol, which is applied to the surface of the sample behind the tool [45,48,51]. The least troublesome and most convenient cooling medium is compressed air because the sample in this case does not require cleaning or drying, and the coolant does not need to be removed from the working area. In addition, air is a high-purity coolant and can be used to process virtually any material. An additional advantage of this method of cooling is the continuous exchange of the cooling agent during the procedure, thanks to which the “cooling parameters” of the air (temperature, flow velocity) do not change during the cooling process, as can be the case with liquid coolants. It should be noted, however, that air has a lower heat capacity than water or cryogenic coolants; hence, the application of compressed air generates lower cooling rates. Nonetheless, it is possible to eliminate this disadvantage and increase the intensity of air cooling, for example, by cooling it with a jet cooling nozzle.

The novelty of the work is an innovative method of cooling the sample during FSP, namely by means of an air stream cooled to −11 °C with a jet cooling nozzle. The proposed solution is located between cooling with compressed air and cooling with cryogenic agents and combines the main advantages of air (cleanliness, no need to remove the coolant from the working area or its disposal/management after treatment) and the advantages of cooling with cryogenic agents (high cooling intensity) [52]. The advantage of the solution used in this study over air cooling results from the much lower air temperature generated by the jet cooling nozzle. To the best knowledge of the authors of this study, there are no similar studies in the world literature on the use of a jet nozzle in FSP, as well as studies on the influence of the angle of the air stream on the size and nature of microstructural changes and material properties. It is worth adding that jet cooling nozzles are offered by suppliers or manufacturers of milling machines as additional equipment for devices; therefore, their availability and potential implementation in industrial conditions is not a problem, which is an additional argument for this solution. The efficiency of the process, the economic effects and the ease of conducting this type of treatment outweigh the previously used technologies of microstructure refinement employed in the process of light metal surface modification using FSP technology.

The main purpose of the work was to analyze the microstructure and properties of the 7075 aluminum alloy subjected to FSP with simultaneous cooling by means of an air stream cooled with a jet nozzle. The reference material was an analogously processed but naturally cooled alloy in still air. The scope of the research also included assessment of the effect of the air blowing method on the size and nature of microstructural changes in the processed zone and the properties of the aluminum alloy.

## 2. Materials and Experiment Procedures

The base material used for the experiment was the 7075 (Al-5.5Zn-2.4Mg-1.6Cu-0.20Cr) aluminum alloy in the T6 state (supersaturated solution treated and artificially aged alloy). This alloy, due to its low density, high specific strength and high fracture toughness, is a material widely applied in many industries, among others, in the production of particularly responsible structures in the aviation and automotive industries [53]. The chemical composition of the aluminum alloy is presented in Table 1.

**Figure 1:**
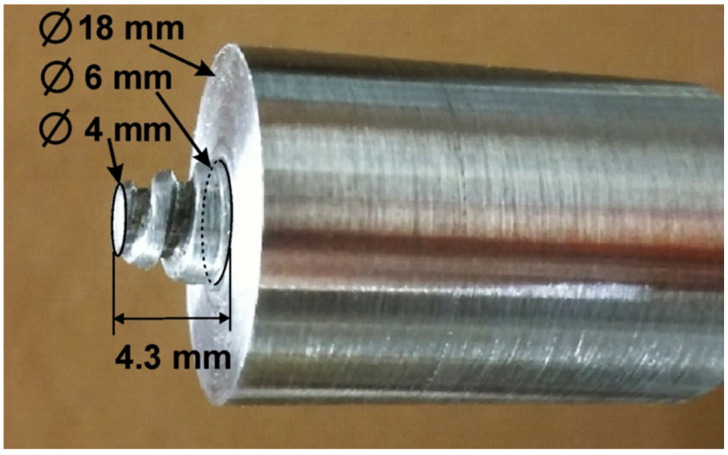
Tool used in friction stir processing of 7075 aluminum alloy.

The material was supplied in the form of 15 mm thick rolled plates. Samples with dimensions of 90 × 70 × 15 mm were cut from the plate, the surface of which was degreased with acetone and then subjected to FSP. Friction stir processing was performed by means of a vertical CNC milling machine (AVIA FNE 50, AVIA S.A., Warsaw, Poland). The tool was made of hardened and tempered X37CrMoV5-1 (H11) hot work tool steel with a hardness of 51 ± 1 HRC. The shoulder diameter of the tool was 18 mm. The tool was equipped with a cone-shaped pin, 4.3 mm long and with diameters of 6 mm (cone base) and 4 mm (cone top), respectively. The side surface of the cone was threaded. The tool utilized in the work is shown in Figure 1. The shoulder tilt angle was fixed at 2°.

**Figure 2:**
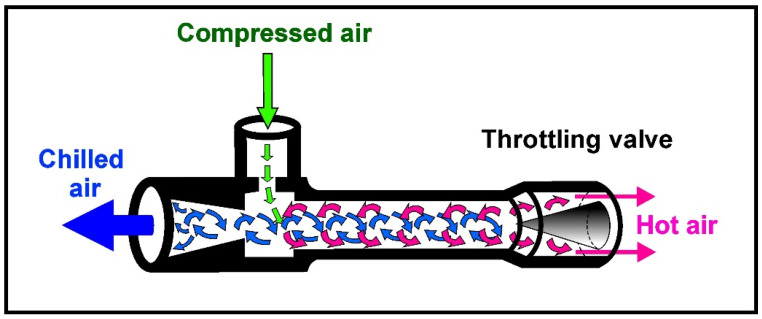
Jet cooling nozzle.

In order to intensify the cooling process of the friction-modified zone, the stand was equipped with an additional cooling system to be employed during processing of the samples. The cooling medium was air-cooled to a temperature of about −11 °C with a jet cooling nozzle. The structure of the nozzle and the principles of its operation are shown in Figure 2.

The jet cooling nozzle is supplied with compressed air, which is set into rotary motion in the nozzle chamber, and then two streams, external and internal, are separated from it. Hot air from the external stream is led outside the nozzle through one of its ends, while the air in the internal vortex loses heat and escapes through the other end of the nozzle in the form of a strongly cooled stream [54]. The temperature of the individual streams depends primarily on the pressure and temperature of the air supplied to the nozzle. The lower the temperature of the supplied air and the higher its pressure, the lower the temperature the air can be cooled to. As a result, the temperature of the cooled air at the outlet can be even several dozen degrees lower than the temperature of the air supplied to the nozzle, which enables high cooling efficiency and energy savings, as the nozzle does not require additional power for its operation. It is worth adding that cooling nozzles are simple, inexpensive and reliable devices that do not require maintenance and can be easily implemented in industrial conditions, and their use does not interfere with the production process. Therefore, nozzles appear to be the ideal solution for spot cooling in FSP and FSW processes.

As part of this work, a commercial cooling nozzle was utilized, offered as an accessory for machine tools, which, according to the manufacturer’s data, can generate a cold air stream with a temperature of up to −46 °C. The auxiliary nozzle, which supplied air previously cooled by the jet cooling nozzle (Exair, Cincinnati, OH, USA), was integrated with the working tool holder, thanks to which its position and distance from the tool and the sample surface were constant during the entire processing. The air pressure supplied to the cooling nozzle was 8 bar.

Two variants of cooling the sample were employed; in the first case, the auxiliary nozzle supplying the cooling stream was inclined at an angle of 45° to the sample surface. This sample was denoted as AC45. The stream of cooled air cooled both the material during friction stir processing and the already modified material located just behind the tool. The cooling stream also cooled the tool shoulder. The distance of the end of the cooling nozzle from the edge of the pin was 20 mm, which allowed for precise directing of the air stream to the place subjected to the friction stir processing, as well as to the tool working in this place. In this way, a double cooling effect was obtained, namely, direct cooling of the modified material by means of an air stream and indirect cooling of the alloy by means of a tool cooled during FSP.

**Figure 3:**
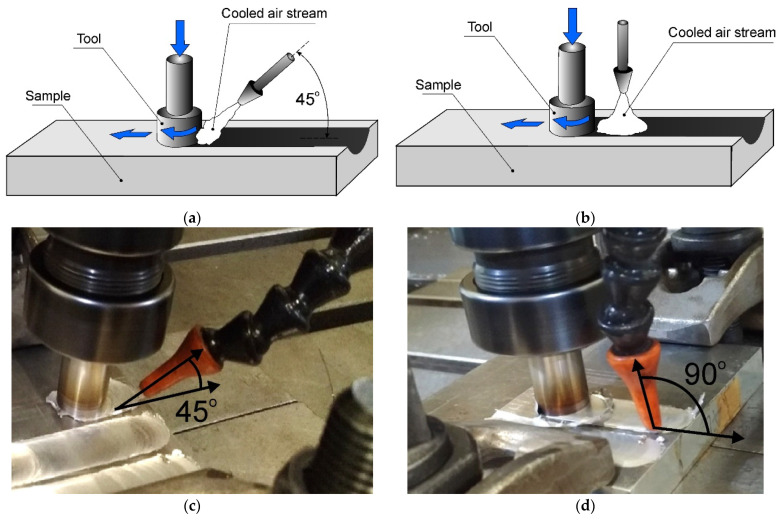
Schemes of FSP variants (**a**,**b**), photos of stands (**c**,**d**).

In the second case, the nozzle was positioned perpendicular to the surface of the sample, and the cooling stream mainly cooled the material located behind the tool, and to a lesser extent, the tool shoulder and the material undergoing frictional treatment at the moment. This sample was denoted as AC90. The FSP variants are shown in Figure 3 in the form of diagrams and photos of the stand. The distance of the end of the cooling nozzle from the edge of the pin in this case was 45 mm, so it was greater than in the case of the AC45 sample. The use of a different distance resulted from the adopted strategy, in the case of the AC90 sample, the focus was on direct cooling of the material behind the pin, i.e., already subjected to friction stir processing, while the indirect cooling of the material with the tool was limited. It should be noted, however, that the key parameter, i.e., the distance of the nozzle tip from the sample surface, was identical in both samples AC45 and AC90, thanks to which the FSP-subjected material was cooled under uniform conditions in terms of air flow parameters.

The use of the different variants in the location of the coolant supply nozzle aimed to demonstrate whether and to what extent a change in the angle of the coolant stream affects the degree of grain refinement in the surface layer of the material. The reference material was an FSPed sample naturally cooled in still air. This sample was denoted as NC.

The detailed processing parameters are presented in Table 2. The selection of FSP parameters was based on the practical knowledge gained during previous tests conducted with the use of 7075 aluminum alloy. The processing was carried out using parameters, at which no previously disqualifying changes in the geometric structure of the surface or unsatisfactory changes in the microstructure of the material were observed.

The obtained samples were subjected to macroscopic and profilometric tests in order to assess the sample surface and analyze its geometric structure. Macroscopic examinations were performed using an Olympus SZ61 stereoscopic microscope (Olympus, Tokio, Japan), and profilometric examinations with a Taylor Hobson TALYSURF 120 profilometer (Taylor Hobson Ltd., Leicester, U.K.). Microstructural examinations were performed by means of an Olympus GX41 light microscope (Olympus, Tokio, Japan) and a JEOL JSM-6610LV scanning electron microscope (JEOL Ltd., Tokio, Japan). The microstructural investigations were carried out on cross-sections perpendicular to the tool traverse direction. The metallographic specimens were etched with Keller’s reagent (2 mL HF + 3 mL HCl + 5 mL HNO_3_ + 190 mL distilled water). To determine the grain size, a measurement technique was used based on determination of the number of grains per unit area (Jeffries method). For each of the samples, the grain size variability was also measured. A representative area located in the near-surface layer was analyzed. Chemical composition analysis was performed using an Oxford Instruments EDS microanalyzer (Oxford Instruments Plc., Abingdon, UK). The hardness was measured on sample cross-sections utilizing a Shimadzu hardness tester (Shimadzu Corp., Kioto, Japan) with a load of 980.7 mN. To evaluate the tribological properties of the material, a laboratory pin-on-disc tribometer, T01-M (ITEE, Radom, Poland), was used under unlubricated sliding contact against a rotating steel ring (HRC 58-63).

## 3. Results and Discussion

### 3.1. Macroscopic and Profilometric Investigations

**Figure 4:**
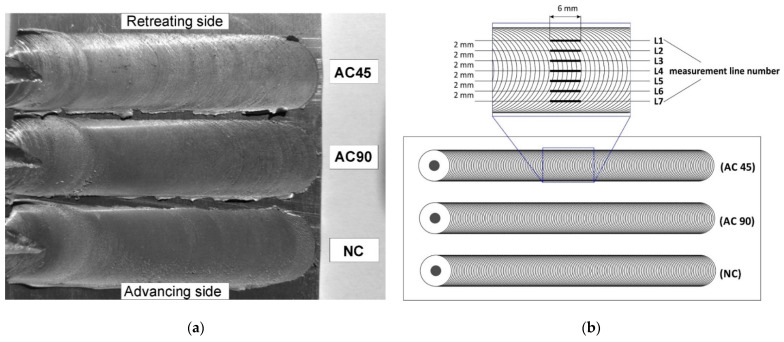
Macroscopic effect of FSP (**a**), scheme for measuring surface roughness of bands (**b**).

The macroscopic effect of FSP is shown in Figure 4a. The main element characterizing the geometric structure of each surface is its roughness, which is the set of irregularities resulting from processing. In order to assess the geometric structure of the samples subjected to surface treatment employing the various FSP variants, three main roughness parameters were measured, namely, the Ra parameter (arithmetic mean deviation of the roughness profile from the mean line), Rz parameter (maximum roughness profile height) and the Rc parameter (mean height of the roughness profile elements). All the samples were subjected to roughness analysis. Seven segment measurements were taken on each band to visualize the variability of the surface geometry at different locations of the band, and in particular to visualize the differences in the values of the roughness parameters in the central part of the band and in the peripheral regions of the band. The measuring section length was 6 mm and the distance between adjacent measuring sections was 2 mm. The adopted measurement methodology is presented in Figure 4b.

**Figure 5:**
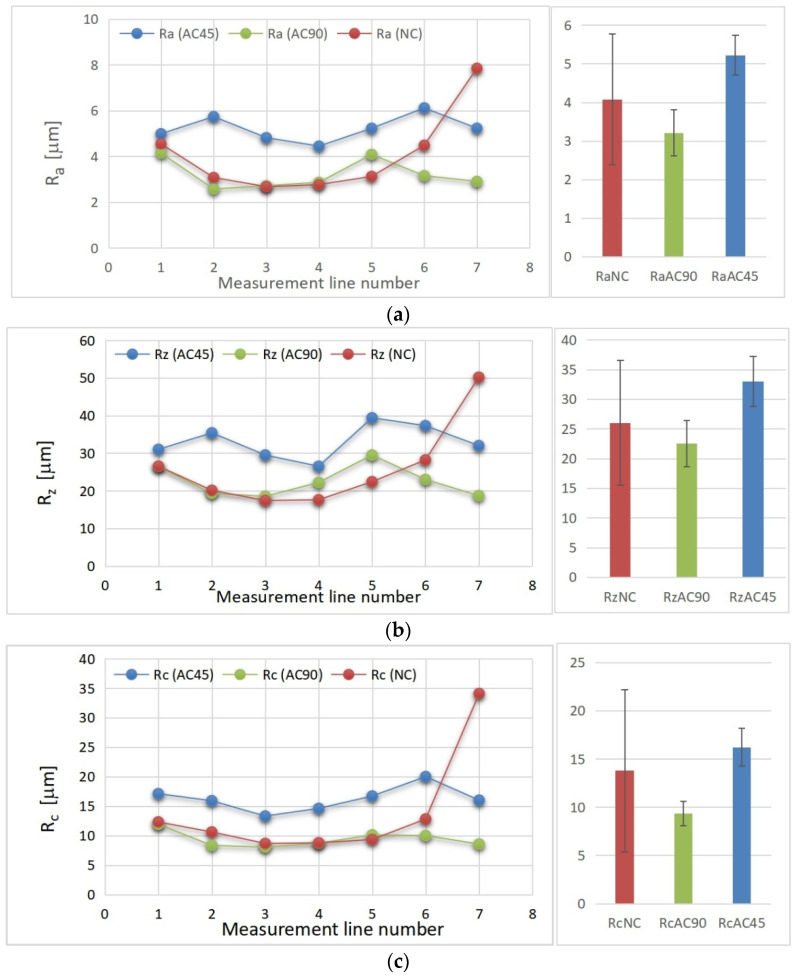
Roughness parameter values: Ra (**a**), Rz (**b**) and Rc (**c**).

The obtained values of the individual roughness parameters as a function of the measuring line location are shown in Figure 5, and the mean values of the Ra, Rz, Rc parameters together with the calculated confidence intervals are given in Table 3.

By analyzing the obtained results, it can be seen that the geometric structure of the AC45 sample was characterized by higher Ra, Rz and Rc parameters than the naturally-cooled sample or the AC90 sample. This fact should be explained by the different kinetics of cooling the material during processing. In the case of the AC45 sample, the cooling stream reduced the thermal effect directly affecting the material at the time of processing, thus influencing the process of shaping the geometric structure of the surface. The lower process temperature resulted in less plasticization of the alloy, which brought about greater surface roughness after FSP. In the case of the AC90 sample, the air stream mainly cooled the material already subjected to FSP, located just behind the pin, and to a lesser extent contributed to a reduction in the thermal effect generated in the tool–material interaction zone and decisive for the degree of plasticization of the material. In this case, the influence of cooling on the process of shaping the geometric structure of the material was smaller because the air stream cooled the material, the geometric structure of which was largely already formed. By analyzing the roughness parameter values as a function of the measuring section location, it can also be noticed that the lowest values of the roughness parameters occurred in the central part of the band and grew as they approached the band boundary, while the rise in the case of the naturally cooled sample was very large on the advancing side. In the case of the remaining samples, no such significant differences were observed on the advancing or retreating side.

The results presented in Table 3 show that the largest spread (the widest confidence interval) relates to FSP without additional cooling (NC) and is due to an unfavorable, strong increase in the tested parameters (Rz, Ra, Rc) on the advancing side. The deterioration of the surface shaping conditions under free cooling conditions (without additional cooling) is an important argument for introducing additional cooling treatments to the friction stir processing of heat-sensitive materials, including the solutions proposed in this article.

### 3.2. Microstructure Evolution

#### 3.2.1. Microstructural Investigations of 7075 Aluminum Alloy in Initial State

**Figure 6:**
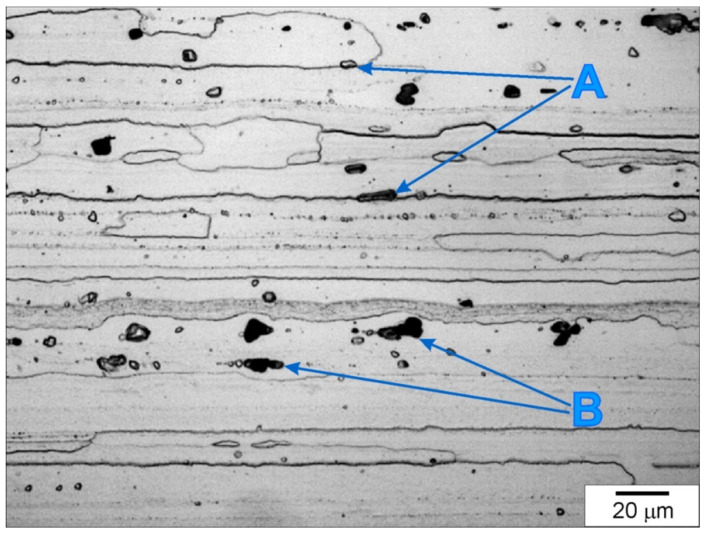
Microstructure of 7075 aluminum alloy in initial state, light microscope, etched specimen.

In order to illustrate the size and scope of changes in the microstructure of the aluminum alloy caused by FSP, detailed studies of the material in its initial state were carried out, paying particular attention to those features and microstructure elements, the evolution of which was to be expected after friction stir processing. The microstructure of the 7075 alloy in its initial state is shown in Figure 6. The 7075 aluminum alloy was characterized by a microstructure typical of materials subjected to directional plastic processing; this microstructure was formed by strongly deformed grains of the α primary solution, with a clearly elongated shape, whose orientation corresponded to the direction of rolling. The average grain length was about 240 µm.

**Figure 7:**
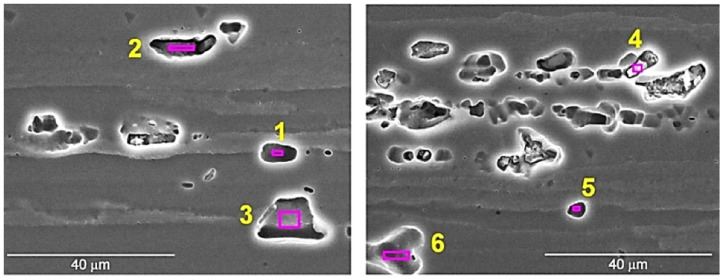
Precipitates of intermetallic phases in 7075 aluminum alloy in initial state, SEM.

**Figure 8:**
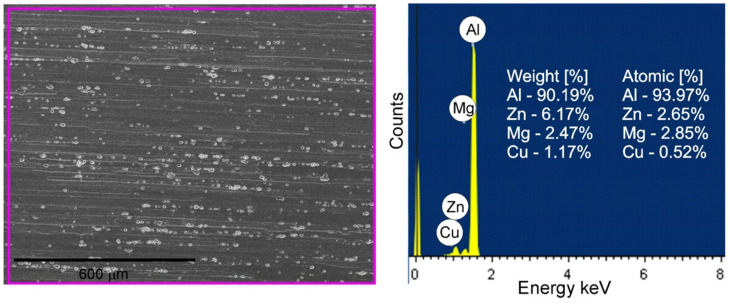
EDS of BM results.

A characteristic feature of the 7075 alloy microstructure was the presence of numerous intermetallic phases located both at the boundaries (examples of precipitates are marked with the symbol A in Figure 6) and inside the deformed grains (examples of precipitates are marked with the symbol B in Figure 6). The precipitates exhibited distinct banding resulting from the applied plastic processing and were present both in the form of clusters and individual particles. The size of the precipitates varied and ranged from approximately 1 µm to 18 µm, with precipitates of dimensions from 4 to 8 µm dominating. The EDS analysis revealed the presence of several different forms of precipitates. The presence of numerous precipitates rich in Al, Zn and Cu was found, as well as numerous precipitates rich in Al, Fe and Cu and less numerous phases rich in Al, Si and Zn, as well as Al, Zn and Mg. The results of the EDS analysis of the exemplary intermetallic phases are presented in Figure 7 and Table 4. The site subjected to EDS analysis is marked with a frame in the figures. Moreover, EDS analysis of a representative part of BM (the base material) was performed in order to compare this composition with the results of the EDS analysis of the material subjected to FSP, which is discussed later in the study. The result of the EDS analysis of BM is shown in Figure 8. The height of the spectral lines of the alloying elements reflects their content in the alloy.

#### 3.2.2. Macro- and Microstructural Investigations of Friction-Stir-Processed 7075 Aluminium Alloy

**Figure 9:**
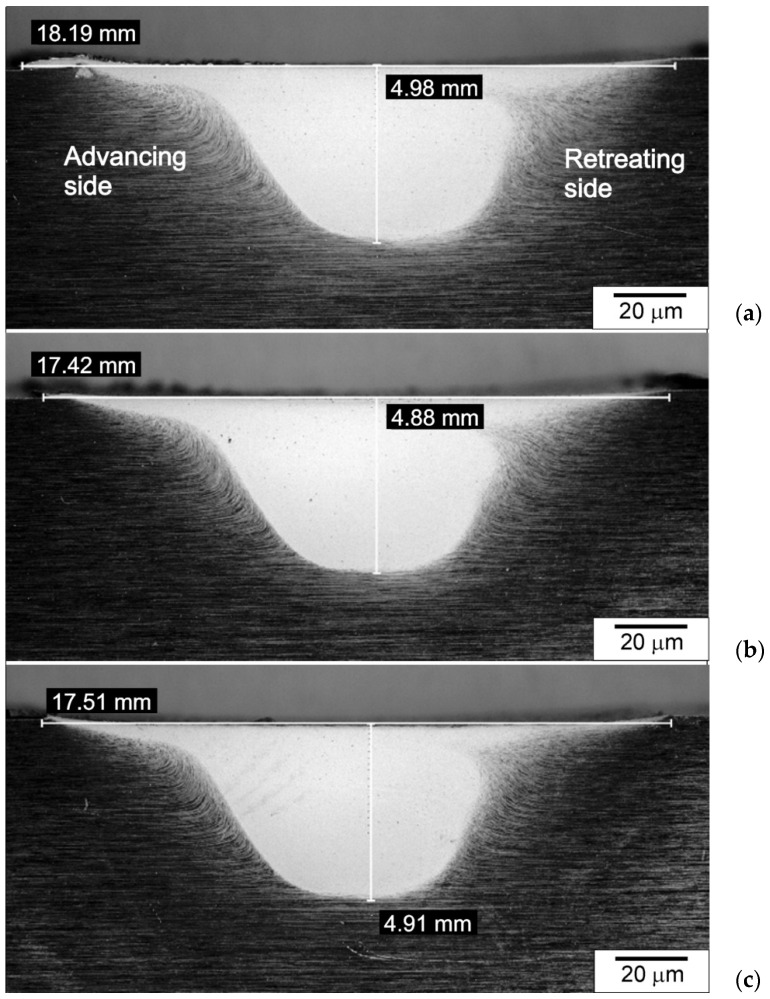
Processed zones of NC (**a**), AC45 (**b**), AC90 (**c**), light microscope, etched specimens.

The friction-stir-processed cross-sections of the samples are shown in Figure 9. The width and depth of the processed zone were measured on each sample. It was found that these dimensions correlate with the diameter of the shoulder of the tool (in terms of bandwidth) and the length of the pin (in terms of the depths of the processed zone). These zones for the samples cooled with the jet nozzle were slightly smaller than for the naturally-cooled samples. This regularity mainly concerned the bandwidth, and to a lesser extent, the depth of the processed zone. This fact should be explained by the differences in the cooling intensity of the samples and the resulting differences in the size of the temperature gradient, which is formed in individual samples. It should be noted, however, that the differences in the dimensions of the zone of microstructural changes were not significant. Differences in the widths of the stirring zone (SZ) between water-cooled and naturally cooled samples were found, among others, by Chen et al. [37]. In the case of using water cooling, the authors registered a reduction in the width of SZ by over 15%.

In Figure 9, the advancing and retreating sides are highlighted. As can be seen, there are clear differences in the shape and course of the processed zone border on the advancing and retreating sides. In the case of the advancing side, the border was more clearly marked than in the case of the retreating side.

The following zones were found in the surface layer of the samples subjected to FSP: the stirring zone (SZ) located in the central part of the microstructural changes zone, the thermomechanically affected zone (TMAZ) and the heat-affected zone (HAZ). The stirring zone was clearly the dominant zone. In this zone, greatly refined grains were present, mostly equiaxed or close to equiaxial in shape. Both the strong grain refinement and the grain shape in SZ prove that dynamic recrystallization (DRX) occurred during FSP, which shaped the microstructure of the material in the stirring zone. Dynamic recrystallization is a predominant mechanism for grain refinement in FSPed alloys. The occurrence of dynamic recrystallization is a consequence of the strong deformation of the material and the influence of high temperature that accompany the treatment. As indicated in the literature, dynamic recrystallization in aluminum alloys can proceed according to various mechanisms, namely, the evolution of grains can be caused by discontinuous dynamic recrystallization (DDRX), continuous dynamic recrystallization (CDRX) or geometric dynamic recrystallization (GDRX) [55]. DDRX is based on the formation of recrystallization grains and the subsequent migration of grain boundaries [56], while CDRX involves the formation of new grains by a gradual misorientation increase of the subgrains [55], while the main evidence for GDRX was related to the few retained high-aspect ratio fibrous grains [55,57]. The mechanism according to which the dynamic recrystallization of the material takes place is a function of the applied FSP parameters, but also of the intensity of the material cooling. This is confirmed by the research of Zeng et al. [55], these authors showed that different DRX mechanisms may occur in aluminum alloys subjected to FSW, depending on the welding parameters and the cooling method used.

The microstructural changes in SZ also prove that the recrystallization temperature was exceeded during FSP, which in the case of aluminum alloys is about 0.6–0.8 Tm, i.e., 300–450°C. In contrast to the SZ, in the thermomechanically affected zone, non-equiaxed grains dominated. This fact proves that the material in TMAZ was plastically deformed, but no recrystallization occurred in this zone because the temperature required for its occurrence was not reached. The non-equiaxed shape of grains in TMAZ is a consequence of lower strains and strain rates, as well as lower peak temperatures experienced by the material in this area [58]. The zone adjacent to the base material was the heat-affected zone (HAZ). In this zone, the material did not undergo plastic deformation, but is only subjected to a thermal cycle. The changes in the microstructure and properties of the material in this zone were only a consequence of the thermal influence.

**Figure 10:**
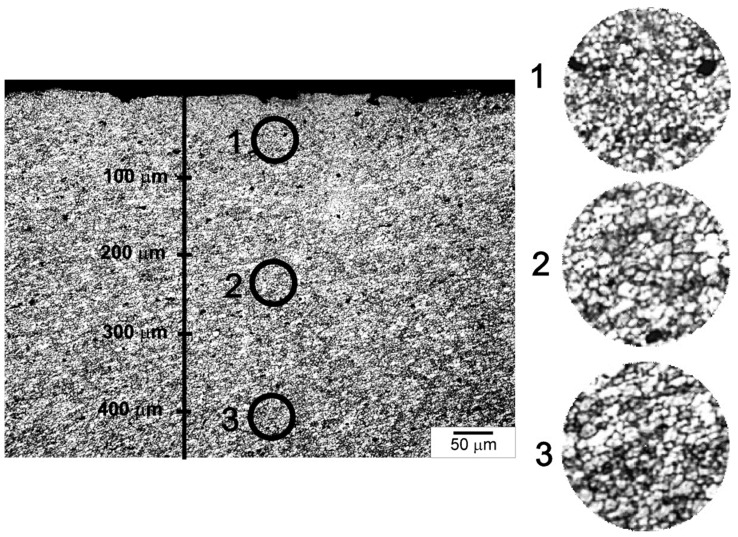
Differences in degree of grain refinement as function of distance from surface. AC90 sample. Sample etched. Light microscopy.

The main measure of the effectiveness of the applied cooling method is, of course, the degree of grain refinement obtained in individual samples. The grain size was measured in the near-surface zone in three areas located at a distance of 0 to 100 μm from the surface, 200 to 300 μm and 400 to 500 μm, according to the diagram shown in Figure 10.

**Figure 11:**
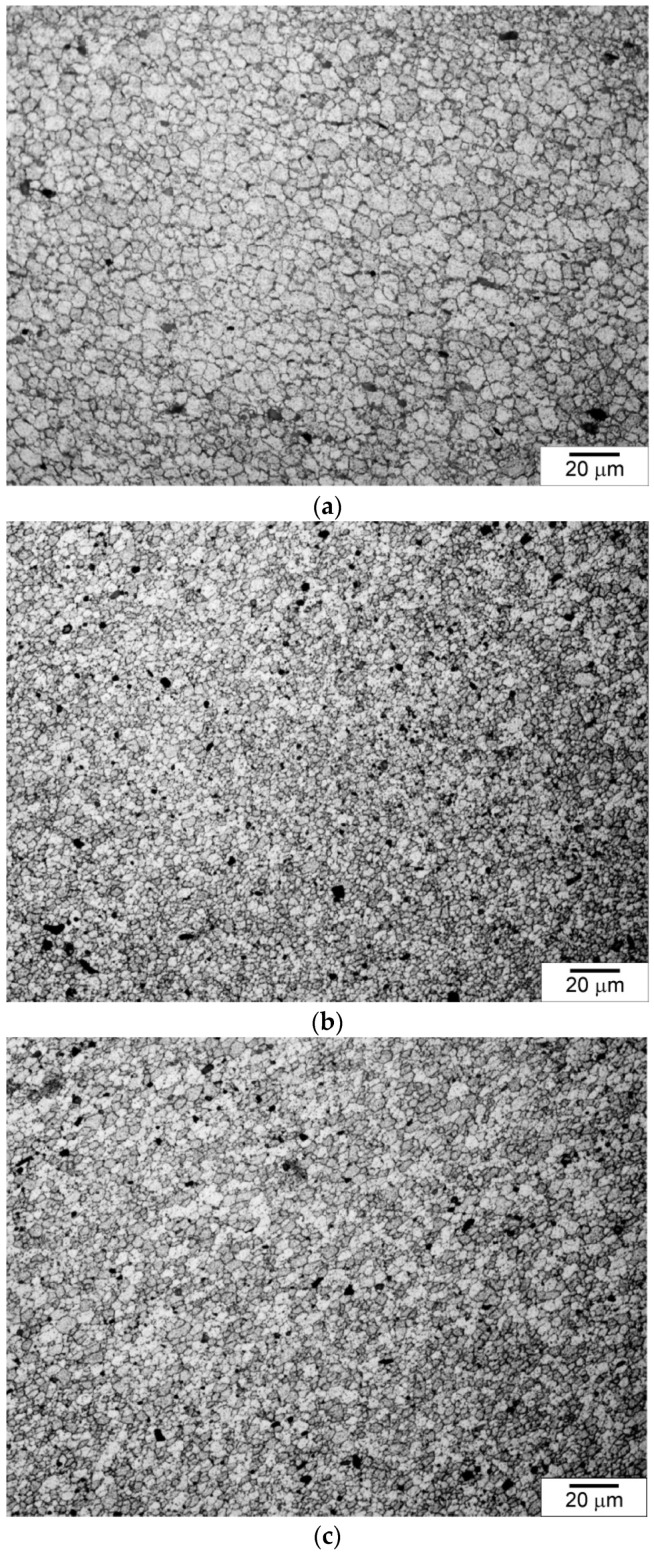
Microstructure of material in near-surface zone: (**a**) NC sample, (**b**) AC45 sample, (**c**) AC90 sample. Light microscope, etched specimens.

**Figure 12:**
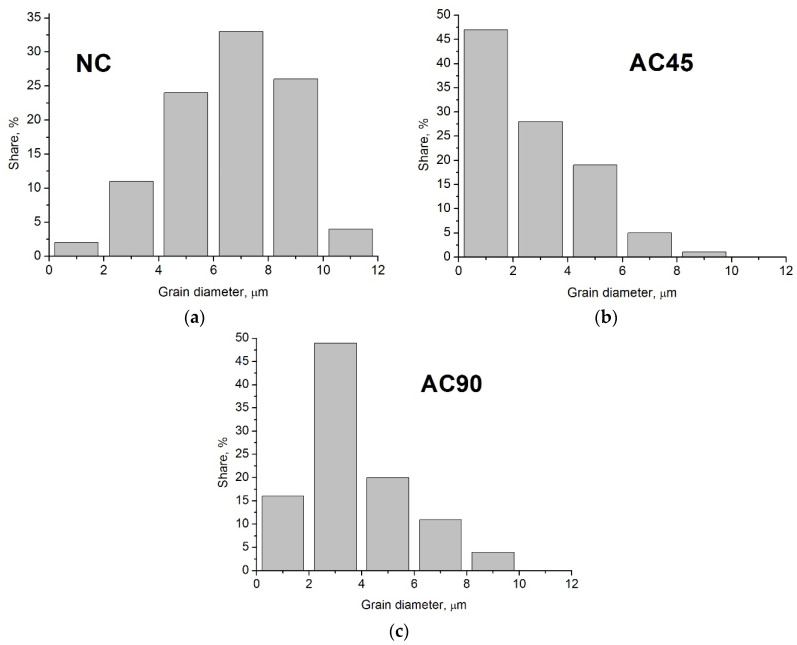
Grain size distributions in NC sample (**a**), AC45 sample **(b**), AC90 sample (**c**).

The microstructural observations revealed that the highest degree of grain refinement occurred in Zone 1, i.e., the zone closest to the surface. The grain sizes in Zones 2 and 3 were similar to each other. In the further discussion, to illustrate the differences in grain size in the different samples, the average grain size determined in the zone closest to the surface, i.e., at a distance of 0 to 100 µm, was used. Examples of microstructures observed in Zone 1 in individual samples are presented in Figure 11, and the grain size distributions are shown in Figure 12. As can be seen, the differences in the grain size are very clear, while in the case of the NC sample the average grain size was about 7.6 µm ± 1 µm, then in the case of the sample cooled with the jet cooling nozzle, the average grain size was 3.2 µm ± 1 µm for the AC90 sample and 1.4 µm ± 0.5 µm for the AC45 sample. Obviously, the degree of grain refinement is influenced by the material cooling rate; when the cooling intensity grows, the number of nucleation sites increases, and consequently, the number of grain boundaries rises, while the grain size decreases.

On the basis of the degree of obtained grain refinement, it can therefore be concluded that the most effective solution is the variant in which the nozzle is positioned at an angle of 45° in relation to the sample surface. It is then possible to obtain the highest degree of grain refinement. It is also worth noting that in this variant, the tool is also cooled, which contributes to an increase in the tool life because then the maximum temperature to which the tool is heated during FSP decreases, and thus the temperature range of the thermal cycle in which the tool works is reduced. This aspect is an additional argument in favor of just such a cooling variant. It should be noted, however, that both variants demonstrated the possibility of obtaining greater grain refinement than with natural cooling. Cooling with a jet nozzle is therefore an effective method of reducing the peak temperature during FSP, which is known to have a significant effect on grain evolution and the final grain refinement of a material. As indicated in the literature, the mechanism of the formation of recrystallized grain depends on the method of cooling and the intensity of cooling the material [55]. The factors which, apart from the cooling rate and the time of exposure to high temperature generated in the material during FSP, also affect the size of the recrystallized grain in the material, are the size of deformation and the rate of deformation of the material, which are a direct consequence of the applied process parameters. It is worth noting that the use of additional cooling also gives the operator the ability to carry out the FSP process with the use of lower traverse speed or higher rotational speeds of the tool. In the absence of additional cooling, both reducing the traverse speed or increasing the rotational speed of the tool would inevitably lead to an unfavorable increase in the grain size in the friction-stir-processed material due to higher heat input and higher peak temperature.

It is also worth adding that regardless of the cooling variant, the use of jet cooling nozzle affects not only the course and dynamics of the processes shaping the microstructure of the material, but also effectively cools the sample after FSP treatment, thanks to which the treatment is carried out in a multi-lane variant, i.e., when a series of bands covering the entire surface of the sample are made, can be performed faster as it is not necessary to cool down the sample after each cycle.

With increasing cooling intensity, the temperature gradient in the material increases, and thus the thermal stresses increase, which in the extreme case may lead to loss of cohesion by the material. In none of the analyzed samples, however, was the presence of microcracks or other material defects found, the presence of which could result from the use of air cooled with a jet cooling nozzle as a cooling agent.

The conducted microscopic examinations revealed high homogeneity of the microstructure of the material and a lack of banding of the precipitates so characteristic of the rolled material. Precipitates were present in the material subjected to FSP, but they were evenly redistributed in the volume of the processed zone and partially dissolved in the alloy matrix. Moreover, no material discontinuities were found in any of the analyzed samples, which is worth emphasizing.

EDS analyses were carried out in order to determine the differences in the chemical composition of the alloy as a function of the distance from the sample surface. The content of the elements was measured in three places, namely in the near-surface zone, in the central part of the processed zone and in the lower part of this zone. The contents of the main elements, i.e., Al, Mg, Zn, Cu, were analyzed, and the results of these analyses are presented in Table 5.

When analyzing the EDS results, one can notice slight differences in the shares of the main alloying elements in relation to their content in BM. The samples treated with FSP using a cooling nozzle had a higher concentration of Zn and Cu in the near-surface layer compared to the content of these elements in the core, with the differences being greater in the case of the AC45 sample. In the case of the NC sample, only a clearly increased concentration of Cu and a lower concentration of Zn in the near-surface zone were found in relation to the content of these elements in BM. According to Pang et al. [59], who analyzed the changes in the microstructure of the AA7075 aluminum alloy subjected to FSP in air and water, the higher concentration of Cu and Zn that was noted in the water-cooled samples may be due to the higher cooling rate, which prevented the redissolution of the alloying elements in the Al matrix. The results of the research carried out as part of this study confirm this hypothesis. It is worth noting, however, that the observed regularities do not apply to Mg. The content of this element in the subsurface zone was either lower than in BM (in the case of the NC samples), or the differences were marginal (in the case of the AC45 and AC90 samples). The analysis of the variability of the content of the main alloying elements as a function of the distance from the surface does not allow unequivocal conclusions to be drawn. While in the case of the AC 45 sample the Zn content clearly decreased with the distance from the surface, in the NC sample this tendency was exactly the opposite, and in the AC90 sample the highest content was found in the central part of the processed zone. There was no clear regularity in the case of Cu content either.

### 3.3. Hardness Measurements

As is known, the grain size has a significant effect on the properties of the material; along with a decrease in the average grain size, the yield strength of the material and the hardness increase, and the plastic properties of the material decline. The influence of the grain size on the hardness *H*_v_ of the material is described by the Hall–Petch equation [60]:

*H*_v_ = *H_o_* + *k*·*d*^−^^1^^/2^

where *H_o_* and *k* are appropriate constants and *d* is average grain diameter.

Since *H*_v_ is proportional to *d*^−1/2^, the finer the grain size is, the greater the hardness value is [61]. Along with the reduction of the grain size, the number of grain boundaries increases, which constitute a barrier to dislocation, and act as pinning points inhibiting further dislocation propagation. This mechanism is the basis of the so-called “grain-boundary strengthening” or “Hall–Petch strengthening”.

Taking into account the Hall–Petch equation and changes in the microstructure of the material caused by the FSP treatment, and especially the strong grain refinement in relation to the starting material, significant growth in the hardness of the material should be expected. On the other hand, the Hall–Petch equation does not take into account the numerous microstructural factors that affect or can affect the hardness of a material. In the case of the 7075 aluminum alloy, such a factor is intermetallic phase precipitates, which strengthen the material and improve its mechanical properties [62]. The potential decrease in hardness of the 7075 aluminum alloy may be a result of the dissolution of the precipitates during FSP. As can be seen from the microstructures presented in Section 3.2.2, a significant number of these precipitates dissolved in the aluminum solid solution during FSP. Nonetheless, the hardness measurement indicated that despite the dissolution of a certain part of the intermetallic phase precipitates, an increase in hardness in the subsurface layer was, nevertheless, noted. Therefore, it can be concluded that the increase in hardness resulting from the strong grain refinement, the disappearance of banding of the precipitates combined with the redistribution of undissolved precipitates effectively compensated for the decrease in hardness resulting from the reduction in the number of intermetallic phase precipitates.

**Figure 13:**
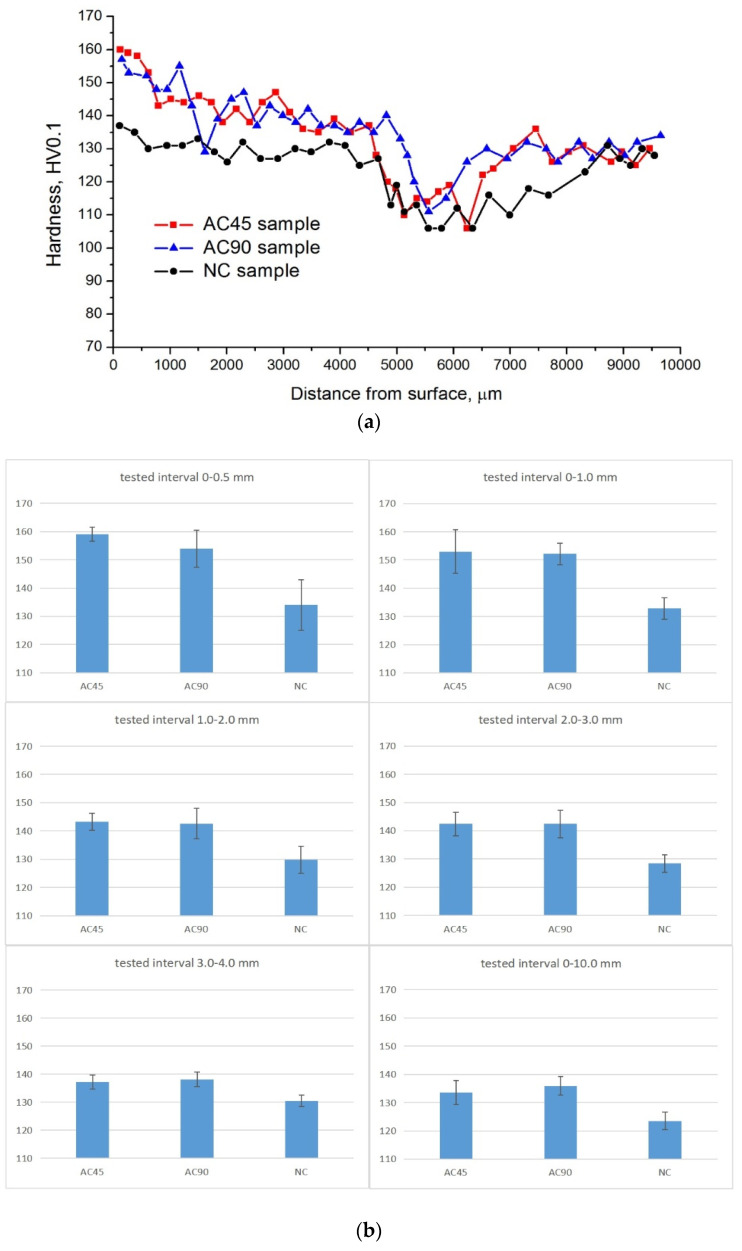
Hardness distributions as function of distance from surface (**a**), graphs of average hardness values and confidence intervals (**b**).

Another key question was whether the use of the jet cooling nozzle contributed to a rise in the hardness of the material in relation to the naturally cooled sample. For this purpose, the average hardness of the material located in the 1 mm thick near-surface zone was calculated. The following hardness values were obtained: 153.7 HV0.1, 151.2 HV0.1, 133.1 HV0.1 for the AC45, AC90 and NC samples, respectively, with an average hardness of BM of about 129 HV0.1. Thus, in the subsurface zone, the hardness of the samples modified with the cooling nozzle in the AC90 variant was higher by about 13.6% compared to the hardness of the naturally cooled material and by about 15.4% in the AC45 variant. In relation to the hardness of BM, the hardness of the AC45 sample was higher by almost 19.2%, and the hardness of the AC90 sample was higher by almost 17.1%. Examples of hardness distributions as a function of the distance from the sample surface are shown in Figure 13, and in Table 6, the average value of hardness as a function of the distance from the processed surface along with the variability range of the examined parameter.

The analysis of the hardness distributions as a function of the distance from the surface shows that the hardness decreases in all samples as they move away from the surface, and this effect is observed to a depth of approximately 5–5.5 mm, then the hardness increases, and its stabilization is observed in the final part of the hardness distribution diagram. Therefore, in the hardness distributions, several ranges can be distinguished, differing in both the amplitude of hardness changes and the level of obtained hardness. The first zone is located at the surface, i.e., to a depth of about 1 mm from it. The material hardness in this zone is the highest and this regularity is observed in all analyzed samples. The high hardness in this zone is of course a consequence of the particularly fine grain refinement, especially in the case of samples cooled with a jet cooling nozzle. It can also be seen that the amplitude of changes in hardness in this zone, expressed by the degree of hardness decrease as a function of the distance from the surface, is clearly greater in the samples cooled with the jet cooling nozzle than in the NC sample. This fact should be explained by the fact that the very intensive cooling obtained thanks to the use of the jet cooling nozzle leads to a very strong refinement of the grain directly at the surface, which results in a clearly higher hardness of the material at this place. However, the scope of the coolant influence is limited, which results in a greater variability in hardness in this zone than in the case of the NC sample. The next zone is an area 1 to about 5–5.5 mm from the surface. In this area, the effect of stabilizing the hardness of the material is visible, the amplitude of changes in hardness in this area is definitely lower than in the near-surface zone, especially in the case of the NC sample, and in the case of AC45 and AC90 samples, a milder decrease in hardness is observed than in the near-surface zone. These results correspond with the results of microscopic tests which showed that in this zone, the grain size was characterized by relatively low variability. At a distance greater than 5–5.5 mm from the surface, i.e., at the boundary of the microstructural changes caused by the surface treatment, a clear drop in hardness to a level below the hardness of the base material can be observed, while in the case of the NC sample, the zone of reduced hardness is clearly wider than in the case of AC45 and AC90 samples, proving that the temperature gradient developed in the material during FSP was smaller, and thus the heat flow rate to BM was smaller. At a distance of more than 6 mm from the surface, an increase in hardness to the level characteristic for base material was observed in all samples. As can be seen, the amplitude of changes in hardness in this zone is relatively small.

By analyzing the literature data on the influence of FSP or FSW on the hardness of the 7075 aluminum alloy, it can be noticed that the increase in hardness is not a regularity. Despite the strong grain refinement favoring the increase in hardness, there is parallel partial dissolution of the precipitate particles, which may lead to a decrease in hardness. The hardness of the 7075 FSPed aluminum alloy is therefore the result of these processes. The lack of a significant increase in the hardness of the 7075 alloy subjected to FSP in relation to the hardness of BM was found, among others, by Sudhakar et al. [63]. Similar effects were also obtained by Kumar et al. [64].

Based on the data presented in Table 6, it can be concluded that with the assumed 95% probability, the unknown hardness falls within the confidence intervals given in this table. It should be stated that the use of additional cooling affects the change of hardness in the entire tested range (0–10 mm) and it is particularly important in the area of the surface layer and zones reaching even 3 mm from the processed surface.

### 3.4. Wear Resistance Tests

**Figure 14:**
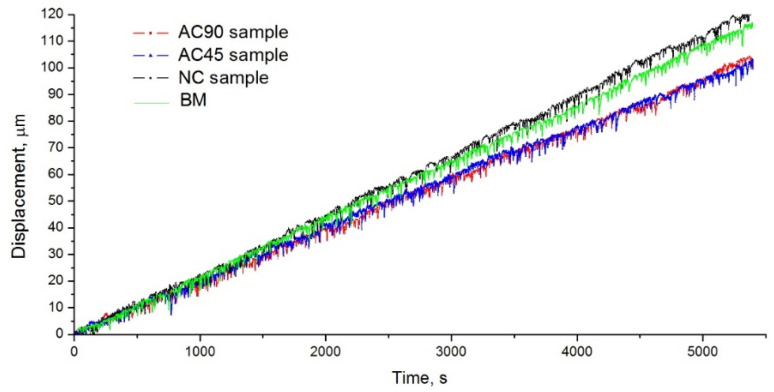
Results of tribological test.

Wear resistance tests were carried out using the pin-on-disc method under dry friction conditions. The specimens for the tribological tests were made from the central part of individual bands. The specimens for the tribological tests had the shape of a cylinder with a diameter of 4 mm and a length equal to the thickness of the sample, i.e., 15 mm. In order to eliminate the influence of differences in the geometrical structure of the surface on the amount of wear of the material, the surface layer, with a thickness of about 25 µm, was removed. This layer was removed with the tribology tester, stopping the abrasion of the specimen when the tester showed a 25 µm linear material loss. Then, the surfaces of the specimens were cleaned of crushed particles and degreased, after which the actual test was started. The test lasted 90 min and was carried out in ambient temperature, which was 23 ± 1 °C. The disc rotation speed was 150 rpm and was identical for all the specimens. The counter-specimen was ring-shaped and made of EN 100Cr6 hard bearing steel (E52100 according to AISI). The wear resistance was determined by measuring the linear wear of the tested specimen. Both friction-stir-processed specimens and the starting material were tested for wear resistance to illustrate the effect of processing on the wear resistance of alloys and possible differences in the wear resistance of the samples modified using different variants of sample cooling. The results of the tribological tests are summarized in Table 7 and presented in Figure 14.

The obtained results prove that the AC45 and AC90 specimens were characterized by a comparable wear intensity, but the wear was slightly lower than that of the analogous naturally cooled material and BM. These results are therefore consistent with the Archard wear equation, according to which the wear rate of the material is inversely proportional to the hardness of the material [65]. Notwithstanding, in the case of the NC specimen, the linear material loss was greater than in the case of BM, even though the hardness of the NC specimen was greater than that of BM. Hence, there was no regularity resulting from the Archard wear equation for the NC specimen. Similar results were obtained, among others, by Gholami et al. [66]; the alloy subjected to FSP analyzed by them was characterized by a lower wear rate than the base material, and the registered wear rates were 1.32 and 1.67 mm^3^·N^−1^·m^−1^, respectively. The lower wear resistance of the NC specimen than BM is mainly a consequence of the partial dissolution of the intermetallic phase precipitates. Although in the case of the AC45 and AC90 samples, there was also a dissolving effect of the intermetallic phase precipitates, the higher degree of grain refinement achieved in these cases largely compensated for the decrease in wear resistance resulting from the reduced number of intermetallic particles.

**Figure 15:**
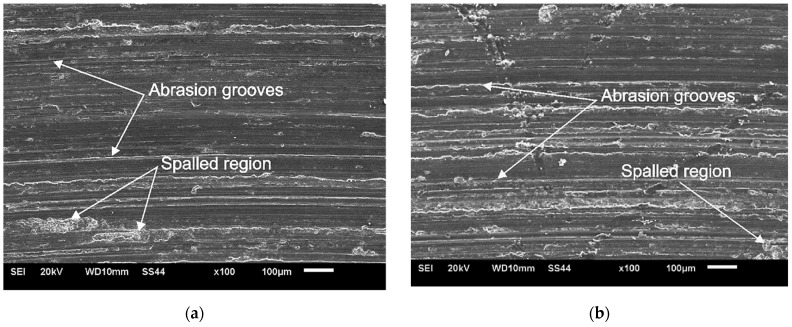

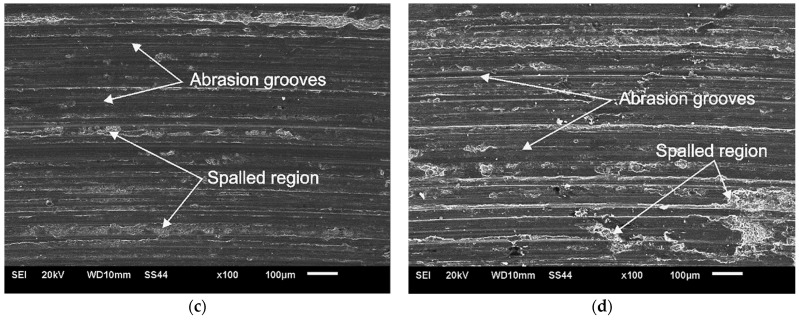
Worn surface morphologies of as-cast specimen (**a**), NC specimen (**b**), AC45 specimen (**c**), AC90 specimen (**d**). SEM.

The samples after the tribological tests were then subjected to SEM examinations in order to visualize their geometric structure and determine the wear mechanism. The conducted observations revealed that the material abraded during the test by micro-cutting and grooving, which proves the abrasive wear of the material in this case. The characteristic scratches and grooves formed on the surface during the tribological test of the material are shown in Figure 15. No significant differences were found in the geometric structure of individual samples subjected to tribological tests. An important parameter useful in the comparative evaluation of the wear resistance of samples with an identical wear mechanism is the width of the grooves. A smaller width of the grooves proves a lesser deformation of the material, and thus also indicates a greater wear resistance of the tested material [67]. SEM tests of the surfaces of NC, AC45 and AC90 samples showed that the width of the grooves in the analyzed samples was similar, therefore, these results correspond to the results of the tribological test, which showed only slight differences in the wear resistance of individual samples. In addition, all the specimens exhibited the local presence of spalled regions, which proves that oxidative wear also occurred during the tribological tests. Under conditions of high friction and the accompanying thermal effect, the risk of oxidation of the sample surface increases, and the intensity of this process also depends on the physicochemical properties of the material, especially its affinity for oxygen. Aluminum is a metal with a strong affinity for oxygen; hence, the likelihood of an oxidation layer on the surface of the sample is very high. It is also worth adding that the oxidized places are more brittle than the metallic substrate; they do not form a permanent bond with the substrate, and therefore can easily detach from the substrate, leaving a characteristic spalled region in the place of detachment.

## 4. Conclusions

The paper presents an analysis of the microstructure and selected properties of the 7075 alloy subjected to FSP with the use of various cooling variants. The novelty of the work is an innovative method of cooling the sample during FSP, namely, by means of an air stream cooled to −11 °C with a jet cooling nozzle. Therefore, an important aspect of the work was also the assessment of the suitability of the applied sample cooling method in shaping the microstructure and material properties.

Microstructural tests and selected material properties showed high efficiency of the new cooling method in relation to cooling in still air, which is expressed by more favorable changes in the microstructure, greater hardness and resistance to wear. The conducted research shows that the use of a jet cooling nozzle, intensifying the cooling process, leads to greater grain refinement, a more homogeneous microstructure and redistribution of intermetallic phase precipitates. The grain size decreases from 240 μm in BM to 7.6 μm in the NC sample and to 3.2 μm and 1.4 μm in AC90 and AC45, respectively. The consequence of microstructural changes caused by processing with air cooling was, in turn, an improvement in hardness, and in the case of the AC45 and AC90 samples, also the abrasion resistance of the material in relation to BM, or the sample subjected to FSP, but modified without employing a cooling system. The application of additional cooling allows the obtained microstructural effects to be influenced, e.g., by changing the position of the cooling nozzle, changing the distance of the nozzle from the frictional site, or changing the air pressure supplied to the cooling nozzle, determining the target air temperature at the outlet of the jet nozzle. The most effective solution is to cool both the surface of the processed material and the tool itself with a stream of air, which was done in the case of the AC45 sample. In the opinion of the authors of this paper, the presented solution has application potential and can be successfully employed in industrial practice, being a competitive solution to the currently used material cooling techniques.

## Figures and Tables

**Table 1:** Chemical composition of 7075 aluminum alloy.

Alloy	Element Content, wt%								
**7075**	**Zn**	**Fe**	**Cu**	**Mn**	**Mg**	**Cr**	**Si**	**Ti**	**Al**
	5.5	0.3	1.6	0.15	2.4	0.2	0.2	0.1	89.55

**Table 2:** Processing parameters of 7075 aluminum alloy.

Parameter	NC	AC90	AC45
Tool rotational speed [rpm]	400	400	400
Tool traverse speed [mm/min]	30	30	30
Plunge time [s]	2	2	2
Plunge speed [mm/min]	6	6	6
Pin length [mm]	4.3	4.3	4.3
Plunge depth [mm]	5.3	5.3	5.3
Auxiliary nozzle inclination angle [°]	–	90°	45°
Distance of end of cooling nozzle from edge of pin [mm]	–	45	20

**Table 3:** Mean values of Rz, Ra, Rc parameters with the determined confidence intervals.

Roughness Parameter	Mean Values			Confidence Interval +/−		
	AC45	AC90	NC	AC45	AC90	NC
Ra	5.23	3.21	4.08	0.52	0.60	1.70
Rz	33.01	22.53	26.06	4.22	3.89	10.57
Rc	16.23	9.37	13.79	1.95	1.27	8.42

**Table 4:** Results of EDS analysis of intermetallic phases.

Element	Mass Fraction of Elements in Intermetallic Phases, wt%					
	No. 1	No. 2	No. 3	No. 4	No. 5	No. 6
Al	80.31	27.30	88.43	44.90	83.55	78.00
Mg	1.93	0.72	2.52	2.03	2.25	1.34
Zn	10.85	3.16	7.94	4.22	11.14	15.33
Fe	–	–	–	12.80	–	–
Cu	3.95	1.20	–	29.92	3.06	5.33
Cr	0.95	–	–	0.34	–	–
O	2.01	32.78	1.11	5.79	–	–
Si	–	34.85	–	–	–	–

**Table 5:** EDS analysis results.

Sample	Element	Mass Fractions of Individual Elements, wt%			Average Value
		Near-Surface Zone	Central Part of SZ	Lower Part of SZ	
NC	Al	89.93	89.91	89.64	89.83
	Mg	2.06	2.32	2.32	2.23
	Zn	5.91	6.15	6.44	6.17
	Cu	2.10	1.62	1.61	1.78
AC45	Al	88.38	89.36	90.32	89.35
	Mg	2.45	2.05	2.18	2.23
	Zn	7.36	6.39	5.85	6.53
	Cu	1.82	2.20	1.66	1.89
AC90	Al	89.97	89.44	90.42	89.94
	Mg	2.47	2.21	2.32	2.33
	Zn	6.26	6.58	5.47	6.10
	Cu	1.30	1.77	1.79	1.62
BM	Al	90.19			
	Mg	2.47			
	Zn	6.17			
	Cu	1.17			

**Table 6:** Mean values of hardness as a function of distance from the surface and ranges of variability of the tested parameter.

Test Interval [mm]	Mean Value			Confidence Interval +/−		
	AC45	AC90	NC	AC45	AC90	NC
0–1 mm	153.7	151.2	133.1	7.8	3.8	3.8
1–2 mm	143.2	142.6	129.8	3.0	5.4	4.8
2–3 mm	142.4	142.4	128.4	4.2	4.9	3.1
3–4 mm	137.2	138.2	130.5	2.4	2.6	2.1
0–max (10 mm)	133.5	135.9	123.5	4.3	3.3	3.1

**Table 7:** Linear losses recorded during tribological test.

Sample	Linear Loss (μm)
BM	116
NC sample	121
AC45 sample	99.6
AC90 sample	100.5

## Data Availability

The data that support the findings of this study are available from the corresponding author, J. Iwaszko, upon reasonable request.
